# *N*^6^-Methyladenosine: a conformational marker that regulates the substrate specificity of human demethylases FTO and ALKBH5

**DOI:** 10.1038/srep25677

**Published:** 2016-05-09

**Authors:** Shui Zou, Joel D. W. Toh, Kendra H. Q. Wong, Yong-Gui Gao, Wanjin Hong, Esther C. Y. Woon

**Affiliations:** 1 Department of Pharmacy, National University of Singapore, 18 Science Drive 4, Singapore 117 543, Singapore; 2 Institute of Molecular and Cell Biology, 61 Biopolis Drive, Proteos, Singapore 138 673, Singapore; 3 School of Biological Sciences, Nanyang Technological University, 60 Nanyang Drive, Singapore 637 551, Singapore

## Abstract

*N*^6^-Methyladenosine (m6A) is currently one of the most intensively studied post-transcriptional modifications in RNA. Due to its critical role in epigenetics and physiological links to several human diseases, it is also of tremendous biological and medical interest. The m6A mark is dynamically reversed by human demethylases FTO and ALKBH5, however the mechanism by which these enzymes selectively recognise their target transcripts remains unclear. Here, we report combined biophysical and biochemical studies on the specificity determinants of m6A demethylases, which led to the identification of an m6A-mediated substrate discrimination mechanism. Our results reveal that m6A itself serves as a ‘conformational marker’, which induces different conformational outcomes in RNAs depending on sequence context. This critically impacts its interactions with several m6A-recognising proteins, including FTO and ALKBH5. Remarkably, through the RNA-remodelling effects of m6A, the demethylases were able to discriminate substrates with very similar nucleotide sequences. Our findings provide novel insights into the biological functions of m6A modifications. The mechanism identified in this work is likely of significance to other m6A-recognising proteins.

All cellular RNAs undergo a range of post-transcriptional modifications, which are important mechanisms employed by nature to increase their structural and functional diversity^1^,^2^. To date, at least 100 chemically distinct modifications have been identified^3^,^4^, among which *N*^6^-methyladenosine (m6A) is currently one of the most important and most intensively studied epigenetic modifications^5^,^6^,^7^. It is universally conserved across all three domains of life, including archaea, bacteria, and eukarya. In eukaryotes, m6A occurs predominantly as internal modification in messenger RNA (mRNA), where it is highly enriched near the stop codon and in the 3′-untranslated regions (3′UTRs)^8^,^9^,^10^. Recent methylome profiling studies in mouse and human revealed that m6A modification is pervasive throughout the transcriptome, and is present in the transcripts of more than 7,600 coding genes and 300 non-coding genes. However, majority of the m6A modifications are uniquely distributed within the DR(m6A)CH consensus motif, where D denotes A, G or U, R denotes G or A, and H denotes A, C or U^11^,^12^,^13^. The exact physiological relevance of m6A remains to be determined, although its widespread occurrence in mRNAs implies important roles associated with the regulation of gene expression and mRNA functions, such as alternative splicing, translation efficiency and mRNA stability^14^,^15^,^16^,^17^. It is also increasingly clear that dysregulation of pathways controlled by m6A modifications may underlie the pathogenesis of a range of human diseases, such as obesity^18^,^19^,^20^,^21^,^22^,^23^, neurological disorders^24^ and, potentially, male infertility^25^.

The m6A landscape is dynamically regulated by a complex interplay between various families of m6A-specific proteins, termed ‘writers’, ‘readers’ and ‘erasers’, which add, interpret and remove the m6A mark, respectively^5^. For instance, the addition of *N*^6^-methyl group on adenosine is catalysed by m6A methyltransferase complexes, such as METTL3-METTL14-WTAP^26^,^27^, and this can be directly reversed by human m6A demethylases FTO (fat mass and obesity-associated protein)^28^ and ALKBH5 (AlkB homologue 5)^25^, both of which are medically-important enzymes belonging to the iron- and 2-oxoglutarate (2OG)-dependent family of AlkB oxygenases (Fig. 1)^18^,^29^. The FTO and ALKBH5 are highly specific for m6A, with little or no activity for other methylated-nucleotides, such as *N*^1^-methyladenosine (m1A, a cytotoxic lesion in DNA), and 5-methylcytosine (m5C, another ubiquitous epigenetic modification found in DNA and RNA)^30^,^31^. Although FTO is also able to demethylate *N*^3^-methylthymidine (m3T; *k*_cat_*/K*_m_ ~0.007 min^−1^μM^−1^) and *N*^3^-methyluracil (m3U; *k*_cat_*/K*_m_ ~0.014 min^−1^μM^−1^), it does so with significantly lower efficiency compared with m6A substrates (*k*_cat_*/K*_m_ ~0.3–0.8 min^−1^μM^−1^) (Figs 1 and 2)^28^,^30^,^32^. Hence, to date, m6A is the only known physiologically-relevant substrate for FTO and ALKBH5. The factors determining their ‘methylated-nucleotide specificity’ remain to be elucidated, although we^30^, and others^29^,^32^,^33^,^34^, have recently shown that at least part of their specificity could be due to distinct structural features within their nucleotide-binding sites and catalytic domains, which enables multiple specific interactions with m6A (Fig. 1). It is also beginning to emerge that adjunct structural elements, such as the nucleotide-recognition lid domain and the L1 loop, which are unique to FTO and ALKBH5, are likely important for their ‘methylated-nucleotide specificity’^34^.

The specificity of FTO and ALKBH5 is not only observed at the nucleotide level, it is also apparent at the transcript level. This is evident from the observation that despite ubiquitous expression of both FTO and ALKBH5 in mammalian cells, only a fraction of m6A sites is found to be demethylated in any given mRNA^24^. Thus the activity of FTO and ALKBH5 is likely transcript-specific, where only certain selected m6A-containing transcripts are being actively demethylated by the enzymes. Consistent with this proposal, FTO and ALKBH5 have highly distinct physiological functions. In particular, FTO has been shown in several studies to be strongly linked with obesity^35^,^36^, while ALKBH5 appears to be essential for spermatogenesis^25^. In order to achieve such distinct biological functions, FTO and ALKBH5 likely target certain mRNA specifically for demethylation.

Conceivably, the substrate specificity of FTO and ALKBH5 may be facilitated through subcellular localisation of the proteins and/or their differential expression in different tissues (FTO is most abundantly expressed in the hypothalamus^18^ whereas ALKBH5 is highly expressed in the testis^25^). It is also possible that the biological system may tolerate some off-target m6A demethylation, particularly in view that m6A is a reversible modification. However, additional mechanisms are likely in place to enable FTO and ALKBH5 to recognise specific m6A-sites.

A number of studies^37^,^38^,^39^,^40^,^41^ have demonstrated that m6A methylation can directly impact the thermodynamic stability and conformations of DNA/RNA. In particular, recent studies by Micura *et al*.^38^ on various self-complementary duplexes showed that m6A has a general destabilising effects on duplex base-pairing, and may promote secondary structure change in certain sequence contexts. The structural influence of m6A is also evident in cellular RNA and appears to be physiologically-relevant. For instance, recent study investigating the structural profiles of RNA in living cells revealed that m6A-modified sites exhibit specific structural signatures; a loss of m6A modifications (through mettl3 knockout) was accompanied by a significant loss of these structural signatures^42^. This observation concurs with transcriptome-wide RNA structural mapping work by Kool *et al*.^39^ where sites adjacent to m6A were generally found to have a strong tendency towards unpaired structure. In a separate study, it was found that m6A modification on MALAT1 (Metastasis Associated Lung Adenocarcinoma Transcript 1; a lncRNA) caused significant alteration to its local structure, which likely serve to facilitate the binding of its regulatory protein HNRNPC^43^,^44^. Inspired by these interesting observations, we envisaged that m6A-induced conformational change could provide a basis for substrate discrimination by m6A demethylases.

Here, we report combined thermodynamic, spectroscopic, gel-shift, thermophoretic, and biochemical studies on the determinants of substrate specificity for human m6A demethylases. Our results reveal that FTO and ALKBH5 do not exhibit strict sequence requirements for substrate specificity, and the highly-conserved GG(m6A)CU consensus motif is, unexpectedly, not a crucial determinant for selectivity. Our results further reveal that m6A serves as a ‘conformational marker’ which dynamically regulates the overall conformation of the modified RNA, and, hence, the substrate selectivity of m6A demethylases. Remarkably, the introduction of m6A modification induces different conformational outcomes in different RNAs sequences, and this profoundly impact their interactions with m6A-recognising proteins, including FTO and ALKBH5. Through the structural message encoded by the ‘m6A mark’, FTO and ALKBH5 are not only able to discriminate substrates with very similar primary nucleotide sequences, but also those that contain the same consensus motif. Our findings, therefore, provide new insights into the biological functions of m6A methylation. The unique recognition strategy identified in this work is likely of significance to other m6A-recognising proteins and, more widely, other RNA-binding proteins.

## Results and Discussion

### Substrate selectivity of FTO and ALKBH5 is not strictly dependent on specific recognition of m6A consensus motif

To date, the substrate preferences and sequence requirements of the m6A demethylases have not been systematically studied. It is not clear if the G(m6A)C and A(m6A)C consensus motifs (where m6A predominantly resides) are essential for substrate recognition. Moreover, besides mRNA, recent studies have also identified m6A modifications in non-coding RNAs, such as transfer RNA (tRNA), ribosomal RNA (rRNA), small nuclear RNA (snRNA) and long non-coding RNA (lncRNA), where they are not found within the same consensus motifs^45^,^46^,^47^. These findings raise the question of whether FTO and ALKBH5 are able to accept m6A on non-consensus sites. Conceivably, other unidentified m6A demethylases may exist which specifically regulate m6A marks on non-coding RNAs.

**Figure 2:**
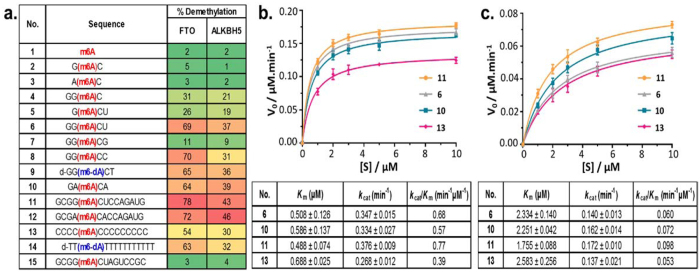
Substrate selectivity and sequence preference of human m6A demethylases FTO and ALKBH5. (**a**) The extent of demethylation of substrates was determined by HPLC after a 1 hour (FTO) or 30-minute (ALKBH5) incubation at 37 °C, pH 7.4. Full length human FTO and catalytically-active human ALKBH5_66–292_ were used. Steady-state kinetics analyses of the demethylation of consensus (**6**, **10** and **11**) and non-consensus m6A substrates (**13**) by (**b**) FTO, and (**c**) ALKBH5. The *K*_m_ and *k*_cat_ values were determined by keeping a constant FTO or ALKBH5 concentration of 0.5 μM. Errors represent S.D. of three replicates.

To explore these interesting questions and to clarify the substrate specificity of FTO and ALKBH5, we analysed the activities of FTO and ALKBH5 against a series of m6A-containing oligonucleotides using a HPLC-based assay (Fig. 2). The m6A substrates investigated consist of short DNAs and RNAs of varying lengths and sequences (Fig. 2a). They are either based on the m6A consensus motifs G(m6A)C (*i.e.* **2**, **4–9**, **11**, **15**) and A(m6A)C (*i.e.* **3**, **10**, **12**), or are based on random sequences (*i.e.* **13**, **14**). We initially determine the minimum substrate length that is required for enzyme recognition. Our results revealed that the m6A nucleotide **1** itself is a very poor substrate for FTO and ALKBH5 (Fig. 2a). The 3-mer core consensus motifs, G(m6A)C **2** and A(m6A)C **3**, also gave negligible demethylation yields (~2–5%), even after prolonged incubation (Fig. 2a). This implies that the residues surrounding the m6A site are likely involved in crucial interactions with the active sites of FTO and ALKBH5. Indeed, the addition of either a guanosine residue at position −2 (relative to m6A in **2** *i.e.* **4**) or a uridine residue at position +2 (*i.e.* **5**) resulted in marked improvements in demethylation yields with both enzymes (~19–31%; Fig. 2a). Interestingly, short 5-mer substrates, such as **6**, **8**–**10** (~64–70%) were found to have similar FTO demethylation yields as that of longer 14-mer substrate **11** (~78%, Fig. 2a), thus the minimum sequence that can be recognised by FTO appears to be only five nucleotides in length. Consistent with these results, kinetic analyses with FTO revealed substantial activity for 5-mer **6** (*k*_cat_*/K*_m_ = 0.68 min^−1^μM^−1^), which is comparable to that of 14-mer **11** (*k*_cat_*/K*_m_ = 0.77 min^−1^μM^−1^) (Fig. 2a,b and Supplementary Fig. S1). ALKBH5 also exhibited a similar activity profile, although it showed a slight preference for **11** (*k*_cat_/*K*_m_ = 0.098 min^−1^μM^−1^) over **6** (*k*_cat_/*K*_m_ = 0.060 min^−1^μM^−1^, Fig. 2a,c and Supplementary Fig. S1). Notably, a guanosine residue at +2 position to m6A is clearly disfavoured, as indicated by the significant reduction in activity for **7** by FTO (~11%) and ALKBH5 (~9%). This concurs with the DR(m6A)CH consensus motif, where ‘H’ is never found to be a guanosine residue. Overall, our results suggest that the short, 5-mer GG(m6A)CU sequence is likely sufficient to define a demethylation site for both FTO and ALKBH5.

We next examine if the m6A demethylases demonstrate any preference for either of the two consensus motifs G(m6A)C and A(m6A)C. Our assay data indicate that FTO and ALKBH5 are able to demethylate GG(m6A)CU **6** and GA(m6A)CA **10** with comparable efficiencies (Fig. 2b,c). In agreement with this result, the demethylation yields for 14-mer G(m6A)C-based substrate **11** were also found to be similar to that for 14-mer A(m6A)C-based substrate **12** (Fig. 2a), implying that m6A demethylases likely do not discriminate between the two consensus motifs. The m6A methyltransferases, on the contrary, strictly favour G(m6A)C over A(m6A)C consensus motifs^48^,^49^, consequently G(m6A)C-based sequences are, depending on species, two- to twelve-fold more abundant than A(m6A)C-based sequences^8^,^9^,^10^.

Our results further revealed that FTO and ALKBH5 do not discriminate between RNA and DNA substrates, as shown by their substantial activities towards both DNA substrates **6**, and its RNA equivalent **9**. Importantly, both FTO and ALKBH5 are able to recognise m6A modifications on non-consensus sites. This is clearly demonstrated by their significant catalytic activities towards the arbitrary RNA sequence **13** (*k*_cat_/*K*_m_ (FTO) = 0.39 min^−1^μM^−1^, and *k*_cat_/*K*_m_ (ALKBH5) = 0.053 min^−1^μM^−1^), which, notably, is only ~2-fold lower than consensus substrate **11** (Fig. 2 and Supplementary Fig. S2). In addition, there are also considerable demethylase activities towards the non-consensus DNA substrate **14** (~63% (FTO), ~32% (ALKBH5), Fig. 2a and Supplementary Fig. S2). These observations are consistent with a recent report which showed that ALKBH5 is able to demethylate the non-consensus sequence rAUUGUCU(m6A)UUGCAGC, although with reduced demethylation yields (~20%)^25^.

Taken together, our results suggest that the GG(m6A)CU consensus motif, while preferred, is not absolutely essential for substrate recognition by FTO and ALKBH5. It is also apparent that FTO and ALKBH5 have the potential for a high degree of promiscuity, although the efficiency of m6A demethylation varies. Hence, sequence information alone is likely insufficient in regulating the substrate specificity of FTO and ALKBH5. We, therefore, propose that additional mechanisms that do not depend exclusively on the specific recognition of the primary nucleotide sequence are likely involved.

### m6A modification destabilises RNA duplexes

We considered the possibility that m6A methylation might modulate substrate specificity of m6A demethylases by fine-tuning the conformation of the modified RNA. Precedence for this possibility comes from observations that conceptually similar epigenetic modifications, in particular *N*-methylation on histone lysine and arginine frequently result in remodelling of chromatin structures, which alters their interactions with DNA^50^,^51^. This is further supported by the number of studies (highlighted in the introduction) which showed that m6A modification can directly impact the secondary structure of RNAs both *in vitro* and *in vivo*^37^,^38^,^39^,^40^,^41^,^42^,^43^,^44^. To the best of our knowledge, to date, m6A has not been explored as a potential determinant of substrate specificity for FTO and ALKBH5.

We begin by studying the effect of m6A modification on RNA conformations. To this end, we employed several 12-mer palindromic RNAs **16**–**23** as model sequences (Table 1 and Supplementary Table S1). Due to their self-complementary nature, they can inherently adopt two main secondary structures in solution, namely (1) duplex conformation, by engaging in intermolecular base pairing, and (2) hairpin conformation, by folding back on themselves. Such structural versatility enables them to mimic the dynamic RNA structures observed under physiological conditions, hence they are particularly suited for our study.

**Figure 3:**
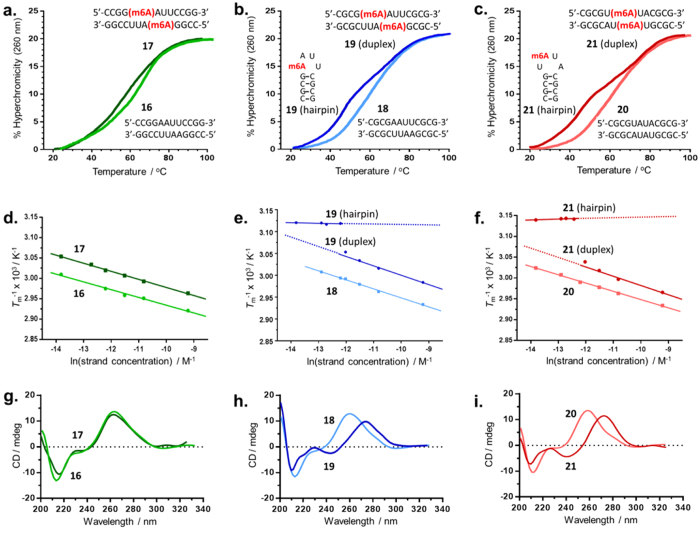
Effects of m6A modification on the overall conformation of model RNA sequences. (**a**–**c**) UV-melting profiles of the methylated RNA sequences and their corresponding reference sequences (colour-coded) at 5 μM strand concentration. The presence of m6A in **19** and **21** triggered a dramatic duplex to hairpin transformation, as indicated by the biphasic melt curves. (**d**–**f**) Van’t Hoff analyses showed dependence of melting temperatures on strand concentrations. At concentrations <5 μM, **19** and **21** gave invariable *T*_m_ of 47.5 °C and 45 °C, respectively due to hairpin formation. (**g**–**i**) Overlay of the CD spectra of methylated and unmethylated pair (both at 5 μM strand concentration) indicates significant conformational change on m6A modification of **18** and **20**.

We first examine the secondary structure of the unmethylated RNA **16** (rCCGGAAUUCCGG) by performing UV-melting analysis under physiologically relevant conditions (*i.e.* in 10 mM sodium phosphate buffer containing 150 mM NaCl, pH 7.4). At a total strand concentration of 5 μM, **16** showed a monophasic, sigmoidal melting profile, indicating the presence of a single structural species (Fig. 3a). In addition, van’t Hoff plot over a concentration range of 1–100 μM **16** revealed melting temperatures (*T*_m_) that are linearly dependent on strand concentration, suggesting that **16** likely exists as a bimolecular duplex structure (Fig. 3d and Supplementary Fig. S3). This is supported by CD analysis of **16**, which shows a dominant positive UV absorption band at 264 nm and a negative absorption band at 212 nm, which are characteristics of an A-form double helix structure (Fig. 3g). The strong hyperchromicity observed in the CD spectrum and UV-melting profiles of **16** further suggests that the duplex structure of **16** is likely to be extensively, if not fully, based-paired.

To determine the structural influence of m6A, we replaced the adenine base at strand position 5 of **16** with an m6A residue, which generated **17** (rCCGG(m6A)AUUCCGG) (Table 1). As with its unmethylated form, **17** was also found to assume an A-form duplex structure, as demonstrated by a monophasic, concentration-dependent melting profile (Fig. 3a,d and Supplementary Fig. S3). In addition, the CD spectrum of **17** superimposes well with that of **16**, implying that m6A modification did not cause any significant conformational change in **17** (Fig. 3g). The presence of m6A, however, did cause an overall destabilisation of the duplex structure of **17** (*T*_m_ = 58.0 °C; 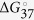 = −13.9 kcal/mol), as indicated by its less favourable free Gibbs enthalpy compared with that of unmethylated duplex **16** (*T*_m_ = 63.0 °C; 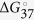 = −15.4 kcal/mol, Table 1). The magnitude of destabilisation caused by a single m6A base is ~1.5 kcal/mol, which is consistent with measurements by others^37^,^38^,^39^,^40^,^41^. In particular, recent work by Kool *et al*.^38^ on multiple sequence contexts indicates that the amount of destabilisation per m6A base ranges from 0.5–1.7 kcal/mol, depending on the level of m6A substitution and sequence contexts. Early study by von Hippel *et al*.^37^ on m6A-containing RNA polymers also indicates a similar level of destabilisation (0.35–0.95 kcal/mol).

Thermodynamic analysis indicates that the observed duplex destabilisation is primarily due to a less exothermic enthalpy (ΔΔ*H*° = 2.5 kcal/mol) which counteracts the favourable change in entropy (ΔΔ*S*° = 2.9 cal/mol/K) (Table 1). In view of the lack of major conformational change between **16** and **17** (Fig. 3g), the introduction of m6A in **17** likely did not cause significant disruption of its duplex base-pairing. Notably, recent NMR studies suggests that m6A likely base pair with U in a Watson-Crick manner in double-stranded RNA^38^. Apparently, this is achieved *via* rotation of the *N*^6^-methylamino group from its energetically preferred *syn*-conformation (with respect to *N*^1^ in the purine ring) to the higher-energy *anti*-conformation. Although this orientation is partially compensated for by hydrogen bonding and Watson-Crick geometry matching with U, the resulting m6A·U base pair is expected to be intrinsically unstable, and this introduces an element of instability in the duplex structure.

### m6A modification discriminates RNA sequences by triggering different conformational outcomes

The above results are interesting because they imply that m6A can, in principle, trigger an overall conformational change in RNA if it occurs on a relatively unstable duplex, where the energy cost to form alternative structures will be relatively low. As a proof of principle, we designed a thermodynamically less stable analogue of **17** by replacing both its ‘CCGG’ segments (rCCGGAAUUCCGG; underlined) with ‘CGCG’, to generate rCGCGAAUUCGCG **19** (*T*_m_ = 55.0 °C; 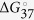 = −12.6 kcal/mol, Table 1). **19** is expected to be thermodynamically less stable compared to **17** due to less favourable nearest neighbour effects^52^.

As anticipated, the UV-melting analysis of **19** revealed an interesting biphasic melting profile, indicating the presence of two structural species under the experimental conditions (Fig. 3b). The first melt transition at *T*_m_ ~47.5 °C has significantly lower hyperchromicity (~14%) than generally observed for a duplex transition, implying a reduced number of base-pairs in this structure. Moreover, van’t Hoff analysis revealed a relatively constant *T*_m_ over 1–5 μM *i.e.* the concentration range at which the first melt transition was detected (Fig. 3e and Supplementary Fig. S4). These observations are indicative of a monomolecular hairpin structure. In contrast, the second melt transition (*T*_m_ ~ 55.0 °C), which dominates the melting profile of **19** at concentrations above 10 μM, showed strong hyperchromicity (~21%) and a concentration-dependent *T*_m_, which are suggestive of a duplex transition (Fig. 3b,e and Supplementary Fig. S4). Hence, **19** likely exists as both hairpin and duplex structures under our experimental conditions. These data concur with the CD spectrum of **19** which showed characteristics of both A-form duplex and B-form hairpin (Fig. 3h).

**Figure 4:**
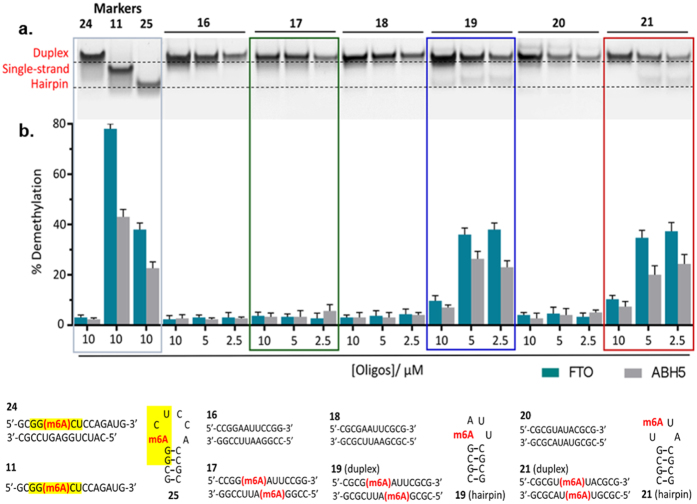
m6A-induced conformational change regulates the substrate selectivity of FTO and ALKBH5. (**a**) Non-denaturing PAGE analysis at 4 °C showed significant hairpin formation in **19** (~20–35% hairpin conversion) and **21** (~30% hairpin conversion) at 5 μM and 2.5 μM strand concentrations, but not in **17** (**b**) This was accompanied by a dramatic increase in the demethylation yields of **19** and **21**, in sharp contrast to **17**. Notably, hairpin formation was not observed for their unmethylated equivalents **16**, **18** and **20**, implying that hairpin formation was triggered by the presence of m6A. RNAs **24**, **11** and **25** are sequences with duplex, random coil and hairpin structure, respectively. The GG(m6A)CU consensus motifs are highlighted in yellow. Errors represent S.D. of three replicates.

We were unable to confirm the presence of hairpin structure with ^1^H NMR study as this species was only formed at ≤5 μM. However, poly-acrylamide gel electrophoresis (PAGE) of **19** under non-denaturing conditions clearly revealed the existence of both duplex (lower mobility band) and hairpin structures (higher mobility band), particularly at low strand concentrations (Fig. 4a). Notably, the ‘hairpin bands’ are inherently faint compared with the ‘duplex bands’, due to the lower number of base-paired sites that can associate with the staining dye. We estimated that at least 20% and 35% of **19** were present as hairpin conformation at 5 μM and 2.5 μM strand concentrations, respectively. Importantly, there was no evidence of hairpin structure in the native PAGE and UV-melting analyses of unmethylated **18** (Figs 3b and 4a). Hence, hairpin formation in **19** was clearly triggered by m6A modification, and likely *via* duplex-to-hairpin conversion.

To obtain additional insights into factors determining m6A-induced conformational change, we further analysed **21** (rCGCGU(m6A)UACGCG), an analogue of **19** where the bases within the centre of the palindrome (underlined) were switched from ‘(m6A)AUU’ to ‘U(m6A)UA’ (Table 1). Interestingly, m6A-induced duplex-hairpin conversion was again observed in the native PAGE analysis of **21**, where similar levels of hairpin formation (~30%) were detected at ≤5 μM (Fig. 4a). This observation was verified by UV-melting and CD analyses (Fig. 3 and Supplementary Fig. S5). The thermodynamic parameters derived from UV-melting experiments suggest that duplex-hairpin transformation in **21** is primarily an entropy-driven process, as apparent from the highly favourable entropy change (ΔΔ*S*° = 86.1 cal/mol/K) (Table 1). However, due to the large enthalpy cost (ΔΔ*H*° = 38.3 kcal/mol), the hairpin form of **21** is only marginally more stable than its duplex form at 37 °C (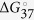 = −1.3 kcal/mol). These data concur with those obtained for the hairpin and duplex structures of **19** (ΔΔ*H*° = 37.5 kcal/mol, ΔΔ*S*° = 86.0 cal/mol/K, 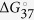 = −1.8 kcal/mol).

Our combined results, therefore, demonstrate that the introduction of a single *N*^6^-methyl group on adenine is sufficient to induce a major overall conformational change in RNA. In addition, the conformational outcomes of m6A modification is highly dependent on sequence context. In RNAs **17**, **19** and **21**, m6A-induced duplex-hairpin transformation was only observed for **19** and **21**, and not for **17**, despite all three RNAs having the same base composition, and highly similar primary nucleotide sequences. Hence, a relatively limited sequence change can have a profound effect on the overall conformation of the RNA, and this may provide a molecular basis for substrate selectivity of m6A demethylases.

### m6A is an important molecular determinant of substrate specificity for FTO and ALKBH5

To understand the role of m6A in regulating the substrate specificity of m6A demethylases, we profiled the activities of FTO and ALKBH5 against **17**, **19** and **21** using a HPLC-based assay. The assay results are summarised in Fig. 4. Overall, the activity profile is highly consistent with the structural influence of m6A on these sequences. In particular, both FTO and ALKBH5 were able to recognise and accept **19** and **21**, but not **17** as substrates, suggesting that their substrate selectivity is indeed, at least partially, regulated by m6A-induced structural change (Fig. 4 and Supplementary Figs S3–S8). In line with this observation, the level of demethylation of **19** and **21** was also found to approximately correlate with the extent of m6A-induced hairpin formation under our assay conditions. At 10 μM strand concentration, where both **19** and **21** were observed to exist predominantly as duplexes, there was little or no demethylation of both substrates by FTO and ALKBH5 (Fig. 4). However, when the concentration was reduced to 5 μM and 2.5 μM, where hairpin formation was appreciable, the demethylation yields of **19** (~45% (FTO), ~25% (ALKBH5)) and **21** (~37% (FTO), ~24% (ALKBH5)) increased significantly (Fig. 4, Supplementary Figs S6 and S7). Moreover, in a negative control experiment, we observed negligible demethylase activity towards the unmethylated analogues **16**, **18** and **20** at all concentrations tested (Fig. 4b). It is thus clear that m6A can critically impact the selectivity of FTO and ALKBH5 by modulating the conformation of m6A substrates. Conceptually, this mechanism could enable FTO and ALKBH5 to distinguish their *bona fide* targets from other potential m6A substrates, including those with very similar primary nucleotide sequences. We further postulate the structural influence of m6A may also facilitate the discrimination of substrates with the same consensus motif.

**Figure 5:**
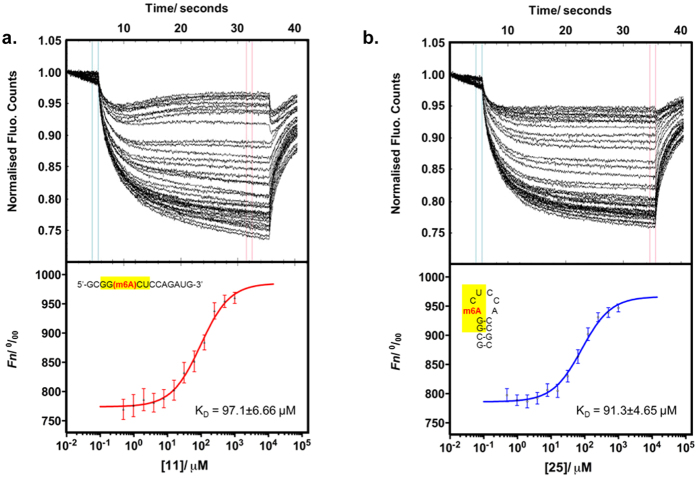
Quantification of binding affinity of FTO to **11** and **25** using MST assay. The normalised time traces (upper panel) and binding curve (lower panel) of FTO with (**a**) **11** (*K*_D_ = 97.1 ± 6.66 μM), and (**b**) **25** (*K*_D_ = 91.3 ± 4.65 μM). Errors represent S.D. of three technical replicates.

**Figure 6:**
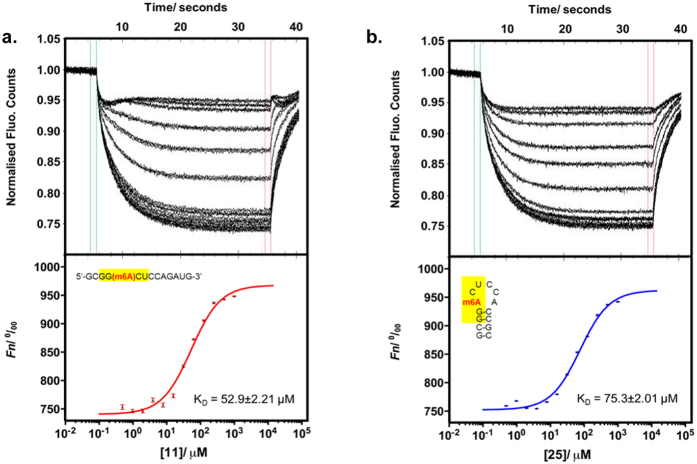
Quantification of binding affinity of ALKBH5 to **11** and **25** using MST assay. The normalised time traces (upper panel) and binding curve (lower panel) of ALKBH5 with (**a**) **11** (*K*_D_ = 52.9 ± 2.21μM), and (**b**) **25** (*K*_D_ = 75.3 ± 2.01 μM). Errors represent S.D. of three technical replicates.

To investigate this possibility, we evaluated the activity of FTO and ALKBH5 against **15** (rGCGG(m6A)CUAGUCCGC), a palindromic substrate containing the GG(m6A)CU consensus motif (underlined). Remarkably, **15** is an extremely poor substrate for both enzymes (demethylation yields ~3% (FTO), ~4% (ALKBH5)) even though it contains the m6A consensus motif. To rationalise this result, we analysed the conformation of **15** and its binding interactions with FTO and ALKBH5. Contrary to other palindromic sequences investigated in this study, such as **19** and **21**, m6A methylation of **15** did not result in any detectable duplex-hairpin conversion. Both **15** and its unmethylated analogue **22** were found to exist almost exclusively as A-form duplex structures, as determined by native PAGE, CD and UV-melting analyses (Supplementary Figs S9–S11). Apparently in its duplex form, **15** showed very poor affinity for FTO and ALKBH5, as demonstrated by biotin-labelled electrophoretic mobility shift assay (EMSA), where there was no detectable binding of biotin-**15** to FTO and ALKBH5, even at 1250-fold excess of proteins (Supplementary Fig. S12). Hence, **15** was not recognised and accepted as substrate by both m6A demethylases. This enables the discrimination of **15** from other substrates containing the same consensus motif, as exemplified by **11** (rGCGG(m6A)CUCCAGAUG) and **25** (rGCGG(m6A)CUCCACCGC) (Fig. 4). In the sequence contexts of **11** and **25**, m6A modification promotes a random coil and hairpin conformations, respectively, both of which are able to bind significantly stronger with FTO and ALKBH5 than duplex **15** (Supplementary Figs S11–S13). Consequently, **11** and **25** are selectively targeted by m6A demethylases (Fig. 4). Results from microscale thermophoresis (MST)-based experiments^53^,^54^ indicate that FTO and ALKBH5 have similar binding affinities for **11** (*K*_D_ (FTO) ~ 97.1 μM; *K*_D_ (ALKBH5) ~ 52.9 μM) and **25** (*K*_D_ (FTO) ~ 91.3 μM; *K*_D_ (ALKBH5) ~ 75.3 μM) (Figs 5 and 6), although their demethylation yields are significantly higher for **11** than **25** (Fig. 4). This implies that the catalysis of hairpin substrate is likely slower compared with single-stranded substrates.

Intriguingly, selectivity for **11** over **15** was also observed for other m6A-binding proteins, in particular the YTH-domain proteins YTHDF2^55^, which showed significant binding with biotin-**11**, but very little or no binding to biotin-**15** (Supplementary Fig. S14). Our combined results revealed that m6A achieves substrate selectivity by regulating the affinity of m6A-recognising proteins for their targets.

## Conclusions

Overall, we use a combination of thermal denaturation studies, CD analyses, gel-shift techniques, microscale thermophoresis measurements and biochemical assays to investigate the factors that modulate the substrate specificity of human m6A demethylases. Consistent with reports by others^37^,^38^,^39^,^40^,^41^,^42^,^43^,^44^, our results reveal that m6A modification has a general destabilising effect on RNA duplexes. We showed that although the magnitude of destabilisation by m6A is relatively small, it could induce a major overall conformational change in certain sequence context. This is clearly demonstrated by oligos **19** and **21**, where the presence of a single m6A modification is sufficient to trigger a remarkable transformation from duplex to hairpin structure.

Importantly, we revealed, through direct biophysical evidence, that such m6A-induced conformational change on RNA (or lack of) could critically influence its interactions with several m6A-recognising proteins, including FTO, ALKBH5 and YTHDF2. This provides, at least partially, a strategy by which these proteins achieve substrate specificity. In particular, through the remodelling influence of m6A, both FTO and ALKBH5 are able to selectively recognise different m6A substrates, including those with highly similar primary nucleotide sequences, as demonstrated by their distinct selectivity for **19** and **21**, over **17**. Intriguingly, this mechanism also likely enables the discrimination of substrates with the same consensus motif, as shown by the lack of activity of FTO and ALKBH5 for **15** compared with **11** and **25**, even though all three sequences contain the GG(m6A)CU consensus motif. Thus m6A likely serves as a ‘conformational marker’ which dynamically regulates the substrate selectivity of m6A demethylases.

Unexpectedly, the highly-conserved GG(m6A)CU consensus motif, which is widely assumed to be essential, is not crucial for substrate selectivity. This is demonstrated by the significant activities of FTO and ALKBH5 towards random sequences **13** and **14** which do not contain the consensus motif. Such apparent lack of a strict sequence requirement by the demethylases is in sharp contrast to other m6A-recognising proteins, such as m6A methyltransferase METTL3^37^,^38^, and m6A-binding proteins YTHDF2^54^, where the GG(m6A)CU consensus motif is strongly preferred.

To our knowledge, this is the first report demonstrating that m6A itself serves as an important selectivity determinant for m6A demethylases. This result is likely of significance to other m6A-recognising proteins and, more widely, other RNA-binding proteins. It is, however, important to appreciate that the identified mechanism alone is unlikely to discriminate m6A modifications on unstructured RNAs. It is also unable to account for differences in specificity between FTO and ALKBH5. Several mechanisms are probably involved which collectively shape the overall selectivity profile of the enzymes. In light of the structure-determining property of the m6A mark^37^,^38^,^39^,^40^,^41^,^42^,^43^,^44^, an interesting question will be whether the physiological functions of m6A demethylases are mediated through dynamic remodelling of their respective RNA transcripts.

## Material and Methods

### Synthesis and purification of DNA/RNA sequences

The DNA/RNA oligonucleotides used in this study were synthesised using standard β-cyanoethyl phosphoramidite chemistry. All synthesiser reagents and phosphoramidites were purchased from Glen Research. In brief, the oligonucleotides were synthesised on a solid support by the automated DNA/RNA synthesiser (Applied Biosystems 394) using a standard 1.0 μmole phosphoramidite cycle of acid-catalysed detritylation, coupling, capping, and iodine oxidation. Cleavage of the oligonucleotides from the solid support and deprotection was achieved by exposure to a 1:1 mixture of 28% aq. ammonium hydroxide and 40% aq. methylamine for 10 min at 65 °C. Deprotection of the 2’-*O*-TBDMS group and initial purification were carried out with Glen-Pak RNA purification cartridges according to the manufacturer’s procedure. The crude products were purified by reverse-phase HPLC using the Waters XBridge OST C18 column (2.5 micron, 10 mm × 50 mm). HPLC solvents used were: solvent A (100 mM triethylammonium acetate buffer, pH 6.5 with 5% acetonitrile) and solvent B (100 mM triethylammonium acetate buffer, pH 6.5 with 15% acetonitrile) with a flow rate of 5 mL/min. All purified oligonucleotides were characterized by MALDI-MS and capillary gel electrophoresis, and were found to be at least 95% pure (Supplementary Table S1).

### Expression and purification of human FTO, ALKBH5 and YTHDF2

Full length human FTO, human ALKBH5_66–292_ and human YTHDF2_385–576_ were expressed and purified as previously reported, with modifications^30^. In brief, all constructs were transformed into *E. coli* BL21 (DE3) Rosetta cells. The transformed cells were grown at 37 °C until an OD_600_ of 0.6 was reached. Protein expression was then induced with isopropyl β-_D_-1-thiogalactopyranoside (IPTG, 0.5 mM, Gold Biotechnology). Cell growth was continued at 16 °C for 16 h, after which the cells were harvested by centrifugation and the resulting cell pellet was stored at −80 °C. The frozen cell pellets were then thawed, resuspended in lysis buffer and disrupted by French Press. Further purification of the protein was achieved using Ni affinity chromatography and gel filtration, as described below. **Full length human FTO** was sub-cloned into pNIC28-Bsa4 to generate a His_6_-tagged FTO_1–505_ construct. FTO in lysis buffer (25 mM Tris, pH 7.5, 500 mM NaCl, 40 mM imidazole and 5 mM β-mercaptoethanol (β-ME)) was purified using Ni affinity chromatography (GE healthcare), followed by gel filtration using HiLoad superdex 200 26/60 (GE healthcare) into the final buffer (25 mM Tris buffer, pH 7.5, 100 mM NaCl, 5% (v/v) glycerol and 5 mM β-ME). **For human ALKBH5**, a His_6_-tagged ALKBH5_66–292_ construct in pNIC28-Bsa4 was used. ALKBH5 in lysis buffer (25 mM Tris, pH 8.0, 500 mM NaCl, 40 mM imidazole and 5 mM β-ME) was first purified using Ni affinity chromatography (GE healthcare), followed by anion chromatography using a 5 mL HiTrap Q HP column (GE healthcare) and gel filtration using HiLoad superdex 75 16/60 (GE healthcare) into the final buffer (20 mM Tris buffer, pH 8.0, 100 mM NaCl and 5 mM β-ME). **Recombinant human YTHDF2**_**385–576**_ was cloned into pGEX-6P-1 from cDNA clones and transformed into *E. coli* BL21 (DE3) Rosetta cells. Purification of YTHDF2 in lysis buffer (50 mM Tris, pH 8.0, 500 mM NaCl, 40 mM imidazole and 5 mM β-ME) was achieved by loading the supernatant to a 5 mL GSTrap^TM^ HP column (GE healthcare). The protein was eluted with gluthione containing buffer (50 mM Tris, pH 8.0, 300 mM NaCl, 10 mM glutathione and 5 mM β-ME). Further purification was achieved by gel filtration using HiLoad superdex 200 26/60 (GE healthcare) into the final buffer (20 mM Tris, pH 8.0, 150 mM NaCl and 5 mM β-ME).

### HPLC-based demethylase assay

The assay was modified from previously reported methods^30^,^56^. The assay was performed in triplicate for each m6A substrate, in a final reaction volume of 25 μL. Reaction consisted of FTO (2 μM) or catalytically active ALKBH5_66–292_ (4 μM), 2-oxoglutarate (300 μM), (NH_4_)_2_Fe(SO_4_)_2_·6H_2_O (150 μM), L-ascorbate (2 mM), m6A-containing DNA/RNA (substrate, 10 μM) in 50 mM HEPES buffer, pH 7.4. The reaction was incubated at 37 °C for 30 min, after which the m6A-containing DNA/RNA was digested by treatment with 1 Unit of nuclease P1 in buffer containing 7 mM of sodium acetate, and 0.4 mM of ZnCl_2_ at 37 °C for 2 h. This was followed by the addition of 1M NH_4_HCO_3_ (1 μL) and alkaline phosphatase (1 Unit). After further incubation at 37 °C for 1.5 h, an internal standard (10 μM, uridine or thymidine for DNA and RNA substrates, respectively) was added to the reaction mixture and the solution was analysed on a HPLC system. The nucleosides were separated using a Zorbax C18 column (4.6 mm × 250 mm) with a gradient of 98% solvent A (MilliQ water + 0.1% TFA) to 100% solvent B (methanol) over 25 min, at a flow rate of 1.0 mL/min at room temperature. The UV detection wavelength was set at 266 nm. Controls without enzyme were also set up. The percentage of demethylation was calculated based on the peak areas of *N*^6^-methyladenine (m6A) in the samples and in their respective controls.

### Steady-state kinetics of m6A demethylation by FTO and ALKBH5

The *K*_m_ and *k*_cat_ values of FTO and ALKBH5 were determined by keeping a constant enzyme concentration of 0.5 μM and varying the substrate concentrations (1, 2, 3, 5 and 10 μM), according to reported methods^30^,^56^. The percentage demethylation at different substrate concentrations was plotted as a function of time (Fig. 2 and Supplementary Fig. S1). The initial velocity (*V*_0_) for each substrate concentration was determined from the slope of the curve at the beginning of a reaction. The Michaelis–Menten curve was fitted using non-linear regression, and the kinetic constants (V_max_, *K*_m_) of the substrate was estimated using GraphPad Prism. All reactions were performed at 37 °C in triplicate and were adjusted to ensure that less than 20% of the substrate was consumed.

### UV-based thermal denaturation studies

The melting of each oligonucleotides was performed on a Cary 3000 UV-Visible Spectrophotometer (Varian) at a total strand concentration of 5 μM (unless stated otherwise) in 10 mM sodium phosphate buffer, pH 7.4 and 150 mM NaCl. Absorbance versus temperature profiles were recorded at 260 nm. The samples were first denatured by heating to 85 °C at 10 °C/min, followed by slow cooling to 20 °C at 0.4 °C/min to ensure a complete annealing of the strands. The melting transitions were then monitored by heating to 85 °C at 0.4 °C/min. To increase the accuracy of measurements, the sixth position was used to record the temperature data points by placing a temperature probe directly in the cuvette. Up to six melting transitions were measured for each oligonucleotide and the average *T*_m_ values were calculated using Varian Cary Software. For sample preparation, lyophilised oligos were reconstituted in the buffer and their concentrations were determined by UV absorbance at 260 nm (A_260_) using a NanoDrop ND-1000 UV-Visible Spectrophotometer. Extinction coefficients were calculated using the nearest neighbour approximation. The extinction coefficient of oligos containing m6A was assumed to be the same as those containing adenosine.

### Analysis of thermodynamic data for bimolecular duplex structures

Each oligonucleotide was measured at six different strand concentrations from 1–100 μM in buffer containing 10 mM sodium phosphate buffer, pH 7.4 and 150 mM NaCl. They were subjected to multiple melting-annealing cycles while monitoring UV absorbance at 260 nm as described above. The melting transitions for duplex structures were assumed to proceed in a two-state manner, and to obey the van’t Hoff’s equation (1) below.


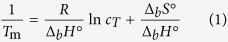


A plot of 1/*T*_m_ versus ln(total strand concentration) gives a straight line, where the slope is R/Δ*H*° and the y-intercept is Δ*S*°/Δ*H*°. Data were fitted using linear least-squares minimisation using GraphPad Prism. The free Gibbs energy (Δ*G*°) were calculated at 37 °C (310.15 K) using the following equation (2).


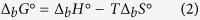


### Analysis of thermodynamic data for monomolecular hairpin structures

The experimental absorbance versus temperature curves were first converted into a fraction of strands remaining hybridized (α) versus temperature curves, which were then fitted to a two‐state transition model using Varian Cary Software.

### Circular dichroism (CD) spectroscopy

CD spectra were obtained with a JASCO J810 spectro-polarimeter. The measurements were carried out with 5 μM oligonucleotides (unless stated otherwise) in a 10 mM sodium phosphate buffer, pH 7.4 and 150 mM NaCl. The oligos solutions were first heated to 90 °C for 5 min, and re-annealed by slow cooling to 4 °C at a rate of 1 °C/min. CD spectra were then recorded in quartz cuvettes (path length 10 mm, 400 μL) from 200 nm to 350 nm using a 10 nm/min scan speed, a spectral band width of 1 nm and a time constant of 4 s. All the spectra were subtracted with the buffer blank and smoothed using the Savitsky-Golay algorithm (polynomial order 10).

### Non-denaturing polyacrylamide gel electrophoresis (PAGE) analysis

Annealed oligonucleotides were loaded to 20% native polyacrylamide gel and electrophoresis was performed at 4 °C in Tris/Borate/EDTA (TBE) running buffer (90 mM Tris, pH 8.3, 90 mM boric acid and 5 mM EDTA). The gel were stained with SYBR^®^ Gold Nucleic Acid Gel Stain and visualized by Gel Dock XR + (Bio-Rad) and Image Lab 4.0 software (Bio-Rad). The fraction of monomolecular hairpin structures was evaluated based on the assumption that the efficiency of the staining of the base pairs in a hairpin was similar to that in a bimolecular duplex.

### Microscale Thermophoresis (MST) Measurement

MST experiments^53^,^57^ were performed on a Monolith NT Label Free system (NanoTemper Technologies) at 25 °C using 40% MST power, and 60% LED power for FTO and 40% LED power for ALKBH5. Laser on and off times were set at 30 s and 5 s, respectively. Zero background standard treated capillaries (NanoTemper Technologies) were used. All experiments were performed in triplicates. Oligos for MST measurement were purchased from Dharmacon^TM^. For determination of binding affinity with FTO, twelve different concentrations of RNA ranging from 1 mM to 490 nM were used. The annealed RNAs were incubated with a mixture of FTO/NiSO_4_ complex (100 nM/1 mM) and NOG (500 μM) in 50 mM Tris buffer (pH 7.5; containing 150 mM NaCl and 0.05% Tween 20) at 25 °C for 20 minutes. Data derived from thermophoresis measurement and temperature-dependent change in fluorescence (T-Jump) were used to determine the binding affinities (*K*_D_) of the RNAs. Curve fitting was performed using the NT Analysis software provided. For experiments with ALKBH5, ALKBH5/MnCl_2_ complex (500 nM/1 mM) was used.

### Electrophoretic mobility shift assay (EMSA)

RNA probes that were labelled at their 3′-ends with biotin were purchased from Keck Foundation Biotechnology Resource Laboratory. Prior to electrophoresis, 2 μL of annealed RNA probes (4 nM final concentration) were incubated with 2 μL of FTO or ALKBH5 or YTHDF2 (0.02, 0.5, 1, 2, 5 μM, and other concentrations as indicated) at 4 °C for 30 min in a binding buffer (10 mM HEPES, pH 7.3, 5% glycerol, 20 mM KCl, 1 mM MgCl_2_, 1 mM DTT and 8 U RNasin^®^ Ribonuclease Inhibitor (Promega)). 20 uL of the RNA-protein mixture was loaded to 7.5% native polyacrylamide gel and electrophoresis was performed at 4 °C for 90 min at 90 V using TBE running buffer. Electrophoresis for ALKBH5 and FTO were performed in buffer without EDTA. Visualisation was carried out using CL-Xposure^TM^ Film (Thermo Scientific), Gel Dock XR + (Bio-Rad) and Image Lab 4.0 software (Bio-Rad).

## Additional Information

**How to cite this article**: Zou, S. *et al*. *N*6-Methyladenosine: a conformational marker that regulates the substrate specificity of human demethylases FTO and ALKBH5. *Sci. Rep.* **6**, 25677; doi: 10.1038/srep25677 (2016).

## Supplementary Material

Supplementary Information

## Figures and Tables

**Figure 1:**
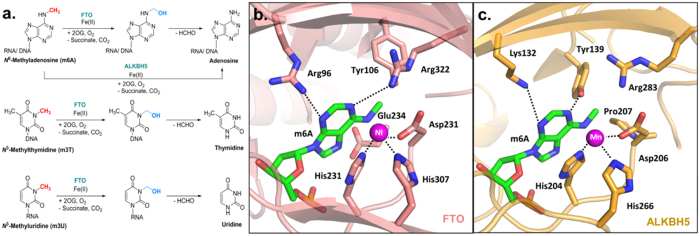
Oxidative demethylation reactions catalysed by FTO and ALKBH5. (**a**) In the removal of m6A mark by FTO, the *N*^6^-methyl group is first oxidised to a hydroxymethyl group, which fragments to give formaldehyde and the demethylated base. ALKBH5 likely catalyses the direct removal of the *N*^6^-methyl group from m6A. m3T and m3U are also demethylated by FTO, but with significantly lower efficiency^28^,^30^,^31^. Superimposition of views from the crystal structure of AlkB-m6A complex (green stick, PDB ID 4NID)^28^ with a structure of (**b**) FTO (salmon residues) (PDB ID 4CXW)^30^, and (**c**) ALKBH5 (orange residues) (PDB ID 4NJ4)^29^. The multiple specific interactions between m6A and the active sites likely enable ‘methylated-nucleotide specificity’.

**Table 1:**
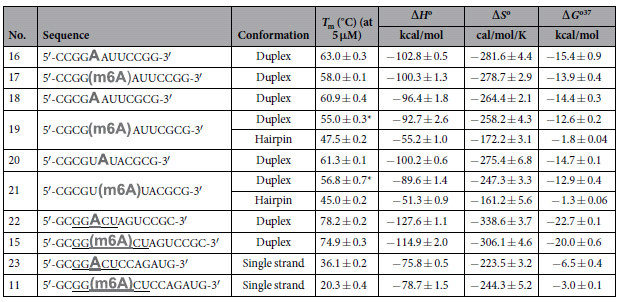
Sequences of RNAs investigated in this study and their thermodynamic parameters.

*T_m_ values were calculated from 1/*T*_m_ versus ln(strand concentration) plot (Supplementary Figs S3–S5 and S9). The GG(m6A)CU consensus motifs are underlined.*T*_m_ was determined at a total strand concentration of 5 μM in a 10 mM sodium phosphate buffer containing 150 mM NaCl, pH 7.4. The thermodynamic parameters for duplex structures were derived from 1/*T*_m_ versus ln(strand concentration) plot, assuming a two-state process. The thermodynamic data for single-strand and hairpin structures were obtained from α (the fraction of strands remaining hybridised) versus temperature plot by curve fitting using Varian Cary software.
