# Supplementary material for: N6-Methyladenosine: a conformational marker that regulates the substrate specificity of human demethylases FTO and ALKBH5
Source: Sci Rep. 2016 May 9;6:25677. doi: 10.1038/srep25677 (PMC4860565; doi:10.1038/srep25677)
Supplement: Supplementary Information [file srep25677-s1.pdf]

Supplementary Information

***N*[6]-Methyladenosine: a conformational marker that regulates the substrate specificity of human demethylases FTO and ALKBH5**

Shui Zou[1], Joel D. W. Toh[1, 2], Kendra H. Q. Wong[1], Yong-Gui Gao[2, 3], Wanjin Hong[2] and Esther C. Y. Woon[1] *

[1] Department of Pharmacy, National University of Singapore, 18 Science Drive 4, Singapore 117 543, Singapore

[2] Institute of Molecular and Cell Biology, 61 Biopolis Drive, Proteos, Singapore 138 673, Singapore

[3] School of Biological Sciences, Nanyang Technological University, 60 Nanyang Drive, Singapore 637 551, Singapore

* To whom correspondence should be addressed. Tel: +65 6516 2932; Fax: +65 6779 1554; Email: esther.woon@nus.edu.sg

Table S1. MALDI-MS data for selected oligonucleotides investigated in this study.

No.	Sequence	MALDI-MS	
		$[M+H]^+$ (calculated)	$[M+H]^+$ (observed)
**16**	5'-CCGG**A**AUUCCGG-3'	3811.4	3811.7
**17**	5'-CCGG**(m6A)**AUUCCGG-3'	3825.4	3825.4
**18**	5'-CGCG**A**AUUCGCG-3'	3811.4	3811.3
**19**	5'-CGCG**(m6A)**AUUCGCG-3'	3825.4	3825.1
**20**	5'-CGCGU**A**UACGCG-3'	3811.4	3811.7
**21**	5'-CGCGU**(m6A)**UACGCG-3'	3825.4	3825.6
**22**	5'-GCGG**A**CUAGUCCGC-3'	4461.8	4461.6
**15**	5'-GCGG**(m6A)**CUAGUCCGC-3'	4475.8	4475.5
**23**	5'-GCGG**A**CUCCAGAUG-3'	4485.8	4485.5
**11**	5'-GCGG**(m6A)**CUCCAGAUG-3'	4499.8	4499.5

(a)

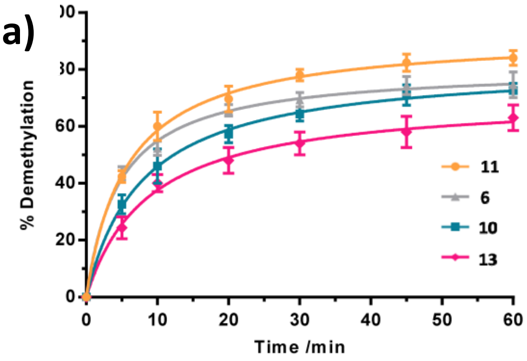

(b)

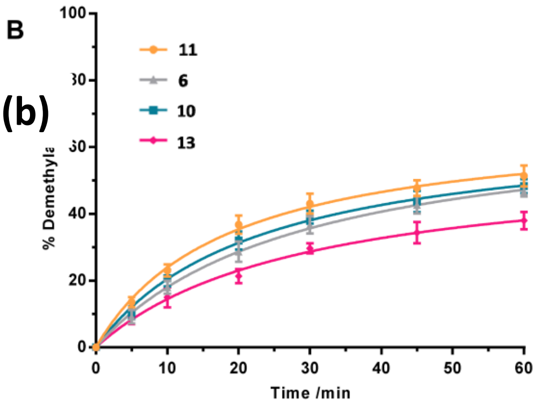

11	GCGG(m6A)CUCCAGAUG
6	GG(m6A)CU
10	GA(m6A)CA
13	CCCC(m6A)CCCCCCCCC

Figure S1. Time course analysis of m6A demethylation activity of (a) FTO and (b) ALKBH5. The percentage demethylation at different substrate concentrations was plotted as a function of time. All reactions were performed at 37 °C, pH 7.4. Errors represent S.D. of three replicates.

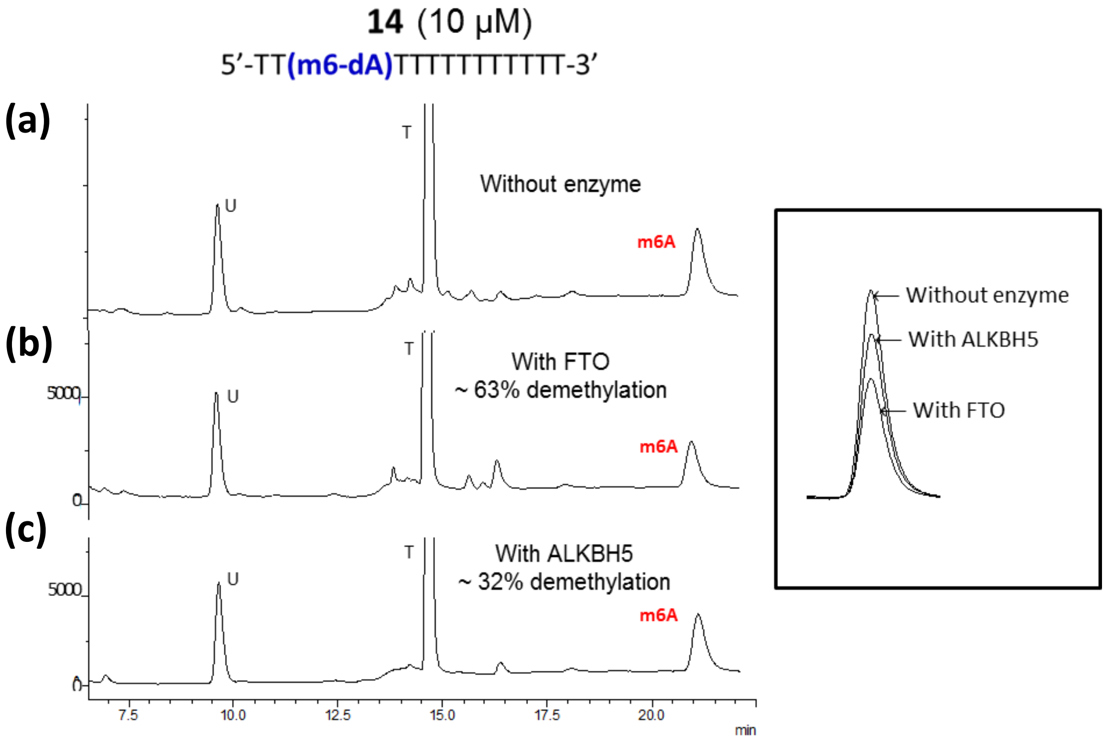

Figure S2. Demethylase activity of FTO and ALKBH5 against non-consensus DNA substrates **14** (10 µM). Representative HPLC traces of digested nucleosides from **14** (a) in the absence of enzyme (control), and (b) after treatment with FTO (2 µM) or (c) ALKBH5 (4 µM). Insert shows an overlay of the m6A peaks from chromatograms A-C. All reactions were performed at 37 °C, pH 7.4 for 30 min (ALKBH5) or 1 hour (FTO). Uridine (10 µM, internal standard) was added to the reaction mixture just before HPLC analysis. Relatively good demethylation yields of 63% and 32% were observed for FTO and ALKBH5, respectively, suggesting that the GG(m6A)CU consensus sequence is not absolutely essential for substrate recognition by FTO and ALKBH5.

(a)

Total strand concentration/ µM	T_m/°C
100	64.3
20	61.0
10	59.4
5	58.0
3	56.5
1	54.3

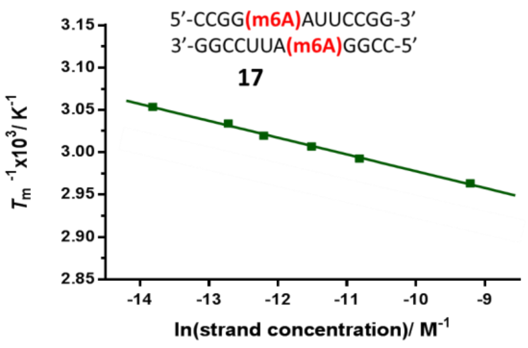

Equation	Y = -0.01982*X + 2.780
R square	0.9974

	T_m/°C (at 5 µM)	$\Delta H°$ kcal/mol	$\Delta S°$ cal/mol/K	$\Delta G°_{37}$ kcal/mol
Duplex	58.0	-100.3	-278.7	-13.9

(b)

Total strand concentration/ µM	T_m/°C
100	69.2
20	65.7
10	64.9
5	63.0
4	61.9
1	59.1

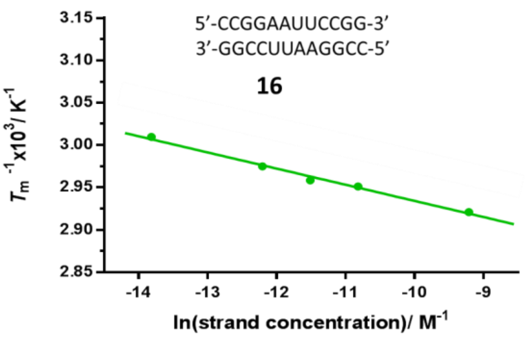

Equation	Y = -0.01934*X + 2.741
R square	0.989

	T_m/°C (at 5 µM)	$\Delta H°$ kcal/mol	$\Delta S°$ cal/mol/K	$\Delta G°_{37}$ kcal/mol
Duplex	63.0	-102.8	-281.6	-15.4

Figure S3. Van't Hoff plot and thermodynamic parameters of (a) methylated RNA **17**, and (b) its unmethylated reference sequence **16** at 1-100 µM strand concentrations in 10 mM sodium phosphate buffer containing 150 mM NaCl, pH 7.4. Both oligos showed dependence of melting temperatures on strand concentrations, implying a bimolecular duplex structure. The thermodynamic data were derived from $1/T_m$ versus ln(strand concentration) plot, assuming a two-state process.

(a)

Total strand concentration/ μM	T_m/°C
100	62.0
20	58.4
10	56.5
6	54.4
5	47.5
3	47.7
2.5	47.4
1	47.3

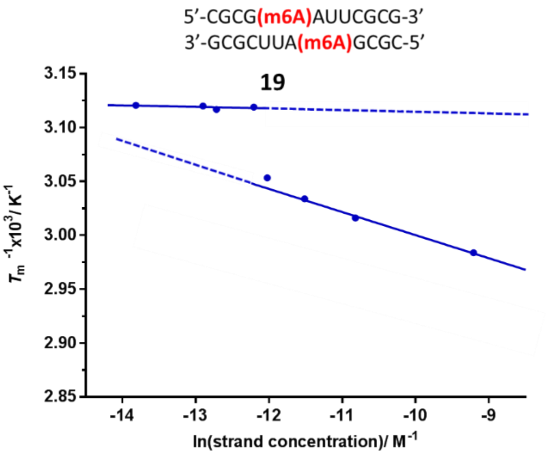

Equation	Y = -0.02144*X + 2.786
R square	0.9962

	T_m/°C (at 5 μM)	$\Delta H°$ kcal/mol	$\Delta S°$ cal/mol/K	$\Delta G°_{37}$ kcal/mol
**Duplex**	55.0*	-92.7	-258.2	-12.6
**Hairpin**	47.5	-55.2	-172.2	-1.8

(b)

Total strand concentration/ μM	T_m/°C
100	67.8
20	64.4
10	62.5
6	61.1
5	60.9
2.5	59.3

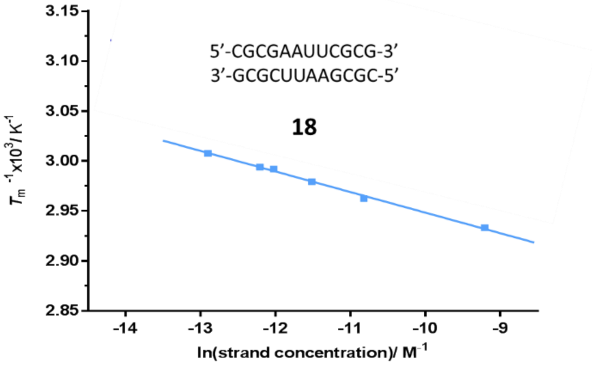

Equation	Y = -0.02061*X + 2.742
R square	0.9965

	T_m/°C (at 5 μM)	$\Delta H°$ kcal/mol	$\Delta S°$ cal/mol/K	$\Delta G°_{37}$ kcal/mol
**Duplex**	60.9	-96.4	-264.4	-14.4

Figure S4. Van’t Hoff plot and thermodynamic parameters of (a) methylated RNA **19**, and (b) its unmethylated reference sequence **18** at 1-100 μM strand concentrations in 10 mM sodium phosphate buffer containing 150 mM NaCl, pH 7.4. **19** showed concentration-dependent melting transition at strand concentrations > 10 μM, implying a bimolecular duplex structure. However, at strand concentration < 5 μM, **19** gave invariable T_m of 47.5 °C, implying the presence of a monomolecular hairpin structure, which is likely formed from the conversion of duplex structure of **19** to hairpin structure on m6A methylation. The thermodynamic parameters for duplex structures were derived from $1/T_m$ versus ln(strand concentration) plot, assuming a two-state process. The thermodynamic data for single-strand

and hairpin structures were obtained from α (the fraction of strands remaining hybridized) versus temperature plot by curve fitting using Varian Cary software. *T_m value was estimated from $1/T_m$ versus ln(strand concentration) plot.

(a)

Total strand concentration/ μM	T_m/ °C
100	64.1
20	60.5
10	58.2
6	55.9
4	45.2
3	45.0
2.5	45.1
1	45.4

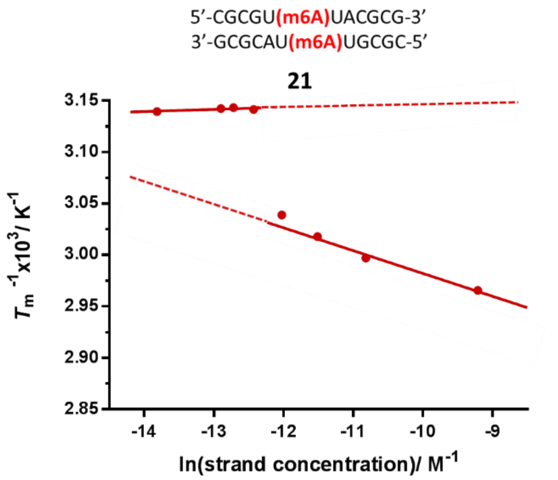

Equation	Y = -0.02218*X + 2.760
R square	0.9884

	T_m/°C (at 5 μM)	$\Delta H°$ kcal/mol	$\Delta S°$ cal/mol/K	$\Delta G°_{37}$ kcal/mol
**Duplex**	56.8*	-89.6	-247.3	-12.9
**Hairpin**	45.0	-51.3	-161.2	-1.3

(b)

Total strand concentration/ μM	T_m/ °C
100	67.7
20	64.4
10	62.7
5	61.3
2.5	59.3
1	57.5

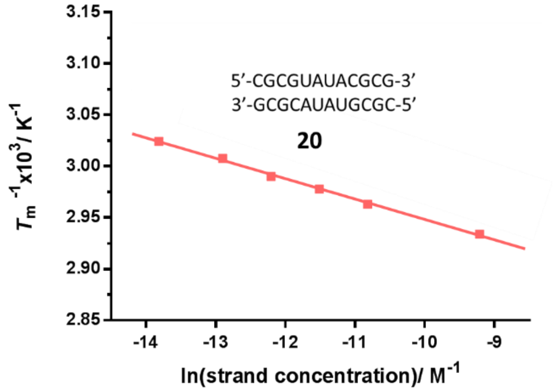

Equation	Y = -0.01984*X + 2.750
R square	0.9977

	T_m/°C (at 5 μM)	$\Delta H°$ kcal/mol	$\Delta S°$ cal/mol/K	$\Delta G°_{37}$ kcal/mol
**Duplex**	61.3	-100.2	-275.4	-14.7

Figure S5. Van't Hoff plot and thermodynamic parameters of (a) methylated RNA **21**, and (b) its unmethylated reference sequence **20** at 1-100 μM strand concentrations in 10 mM sodium phosphate buffer containing 150 mM NaCl, pH 7.4. **21** showed concentration-dependent melting transition at strand concentrations > 10 μM, implying a bimolecular duplex structure. However, at strand concentration < 5 μM, **21** gave invariable T_m of 45.0 °C, implying the presence of a monomolecular hairpin structure, which is likely formed from the conversion of duplex structure of **21** to hairpin structure on m6A methylation. The thermodynamic parameters for duplex structures were derived from $1/T_m$ versus ln(strand concentration) plot, assuming a two-state process. The thermodynamic data for single-strand

and hairpin structures were obtained from α (the fraction of strands remaining hybridized) versus temperature plot by curve fitting using Varian Cary software. *T_m value was estimated from $1/T_m$ versus ln(strand concentration) plot.

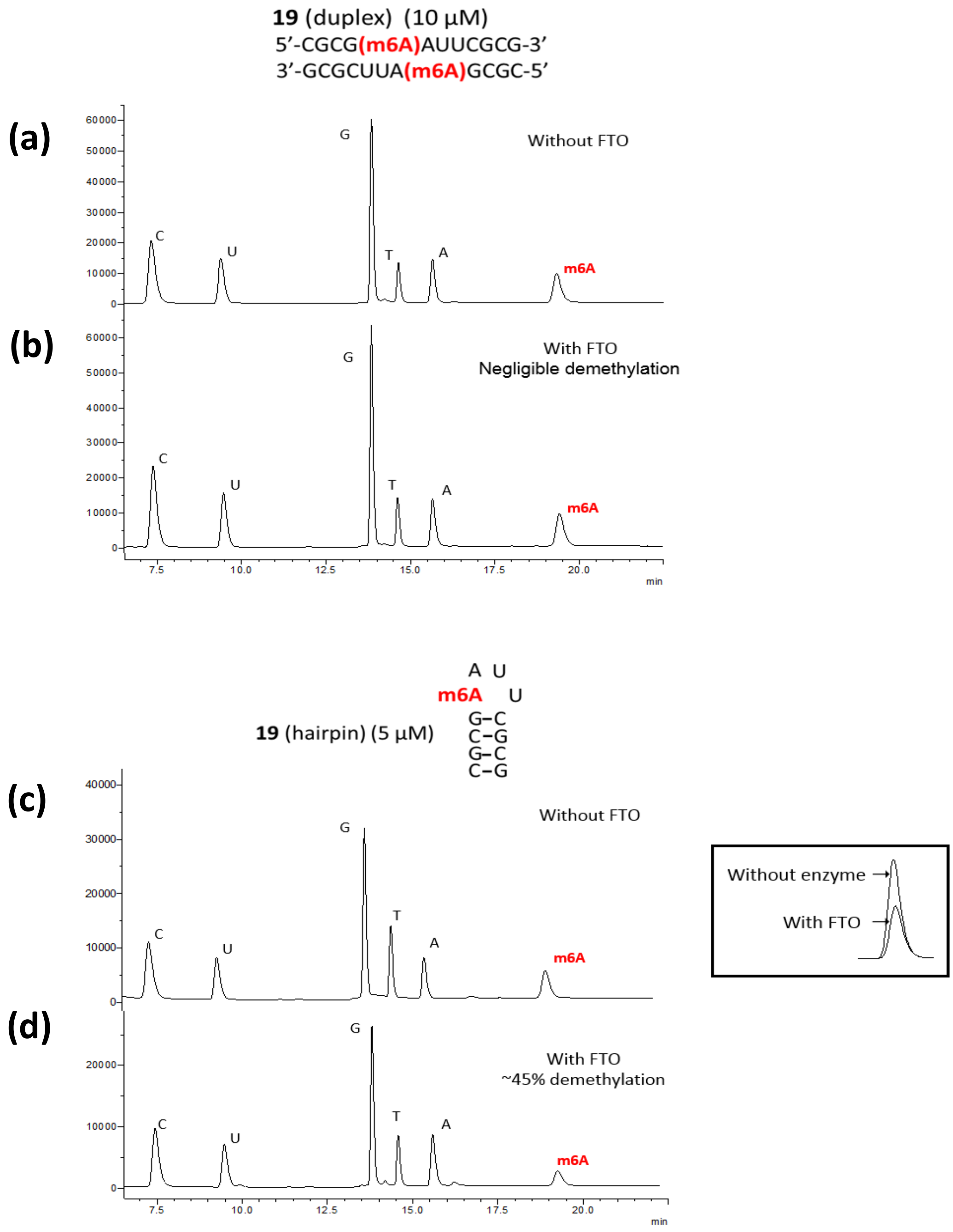

Figure S6. Representative HPLC traces of digested nucleosides from **19** after treatment with FTO (2 µM) at 37 °C, pH 7.4 for 1 hour. (a-b) At strand concentration of 10 µM, **19** existed predominantly as duplex structure, this resulted in little or no demethylation by FTO. (c-d) At lower strand concentration of 5 µM, there is significant hairpin conversion, resulting in a dramatic increase in demethylation yield (~45%). Insert shows an overlay of the m6A peaks from chromatograms c and d. Thymidine (10 µM, internal standard) was added to the reaction mixture just before HPLC analysis.

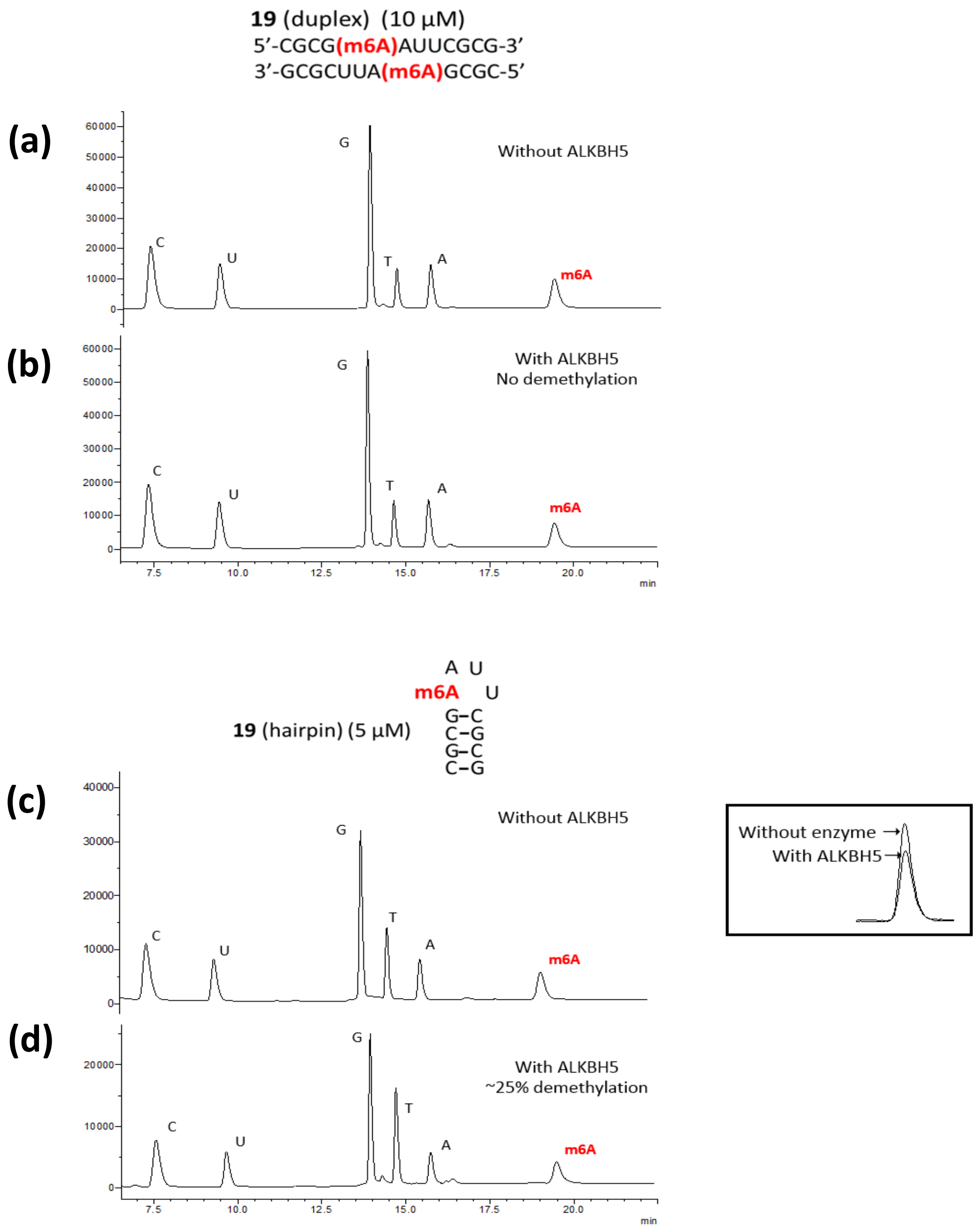

Figure S7. Representative HPLC traces of digested nucleosides from **19** after treatment with ALKBH5 (4 µM) at 37 °C, pH 7.4 for 30 min. (a-b) At strand concentration of 10 µM, **19** exist predominantly as duplex structure, this resulted in little or no demethylation by FTO. (c-d) At lower strand concentration of 5 µM, there is significant hairpin conversion, resulting in a dramatic increase in demethylation yield (~25%). Insert shows an overlay of the m6A peaks from chromatograms c and d. Thymidine (10 µM, internal standard) was added to the reaction mixture just before HPLC analysis.

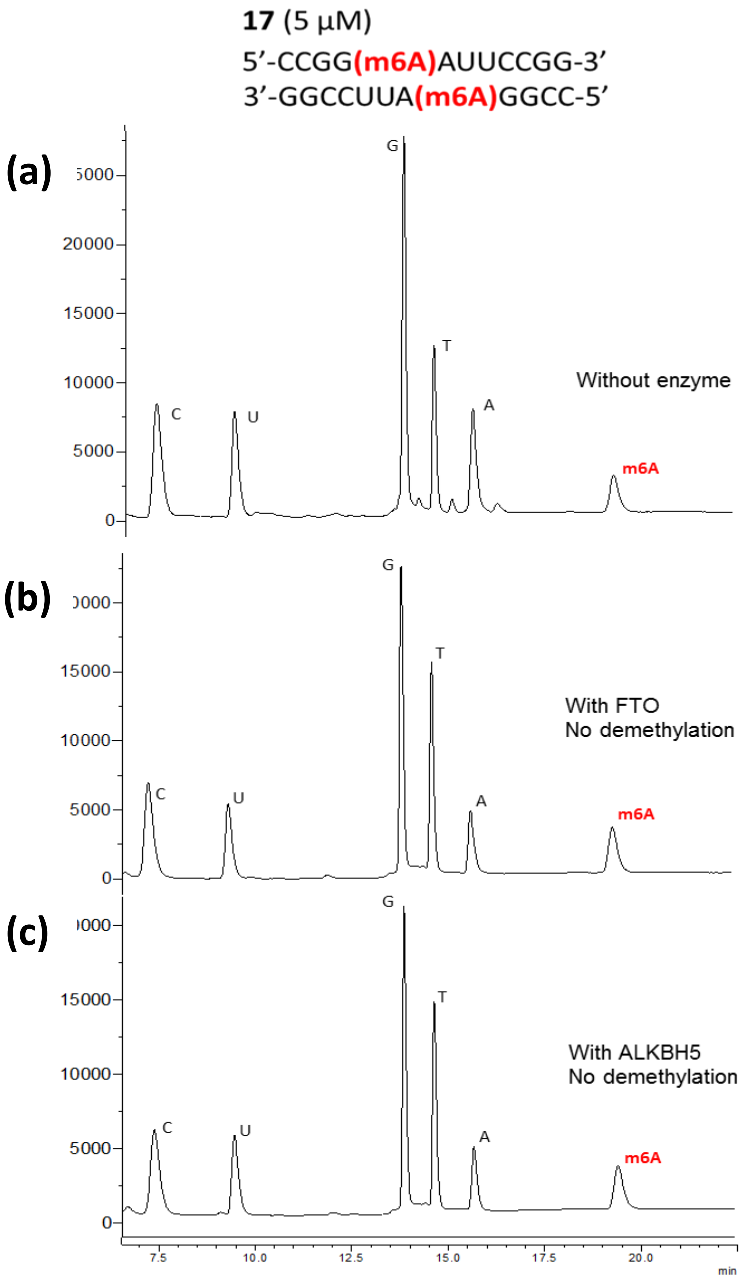

Figure S8. Demethylase activity of FTO and ALKBH5 against RNA substrate **17** (5 µM). Representative HPLC traces of digested nucleosides from **17** (a) in the absence of enzyme (control), and (b) after treatment with FTO (2 µM) or (c) ALKBH5 (4 µM). All reactions were performed at 37 °C, pH 7.4 for 30 min (ALKBH5) or 1 hour (FTO). Thymidine (10 µM, internal standard) was added to the reaction mixture just before HPLC analysis. There was little or no demethylation of **17** by FTO and ALKBH5, even though **17** has very similar primary nucleotide sequence as **19**. Hence substrate selectivity of FTO and ALKBH5 is dependent on the structure-determining effect of m6A modification.

(a)

Total strand concentration/ µM	T_m/°C
20	77.2
10	76.1
5	74.9
4	74.1
3	73.8
1	70.9

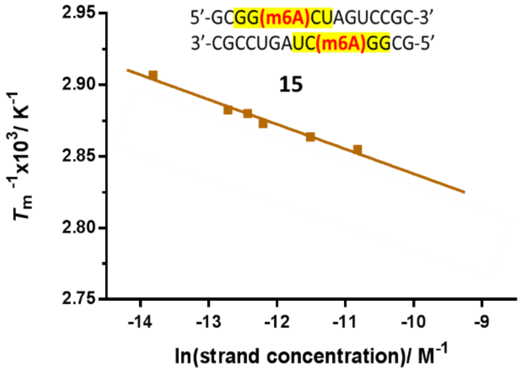

Equation	Y = -0.01730*X + 2.665	
R square	0.9811	

	T_m/°C (at 5 µM)	$\Delta H°$ kcal/mol	$\Delta S°$ cal/mol/K	$\Delta G°_{37}$ kcal/mol
Duplex	74.9	-114.9	-306.1	-20.0

(b)

Total strand concentration/ µM	T_m/°C
20	81.5
10	80.0
5	78.2
4	77.9
3	77.3
1	75.9

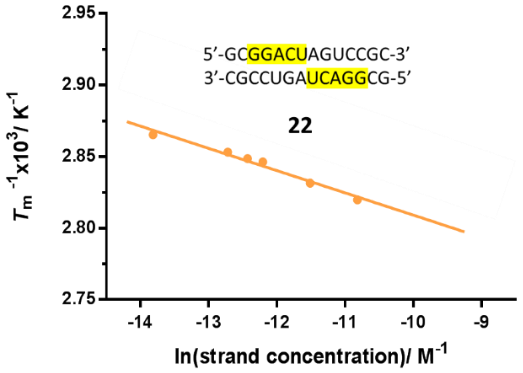

Equation	Y = -0.01557*X + 2.653	
R square	0.9771	

	T_m/°C (at 5 µM)	$\Delta H°$ kcal/mol	$\Delta S°$ cal/mol/K	$\Delta G°_{37}$ kcal/mol
Duplex	78.2	-127.6	-338.6	-22.7

Figure S9. Van't Hoff plot and thermodynamic parameters of (a) methylated RNA **15**, and (b) its unmethylated reference sequence **22** at 1-20 µM strand concentrations in 10 mM sodium phosphate buffer containing 150 mM NaCl, pH 7.4. Both oligos showed dependence of melting temperatures on strand concentrations, implying a bimolecular duplex structure. The thermodynamic data were derived from 1/T_m versus ln(strand concentration) plot, assuming a two-state process.

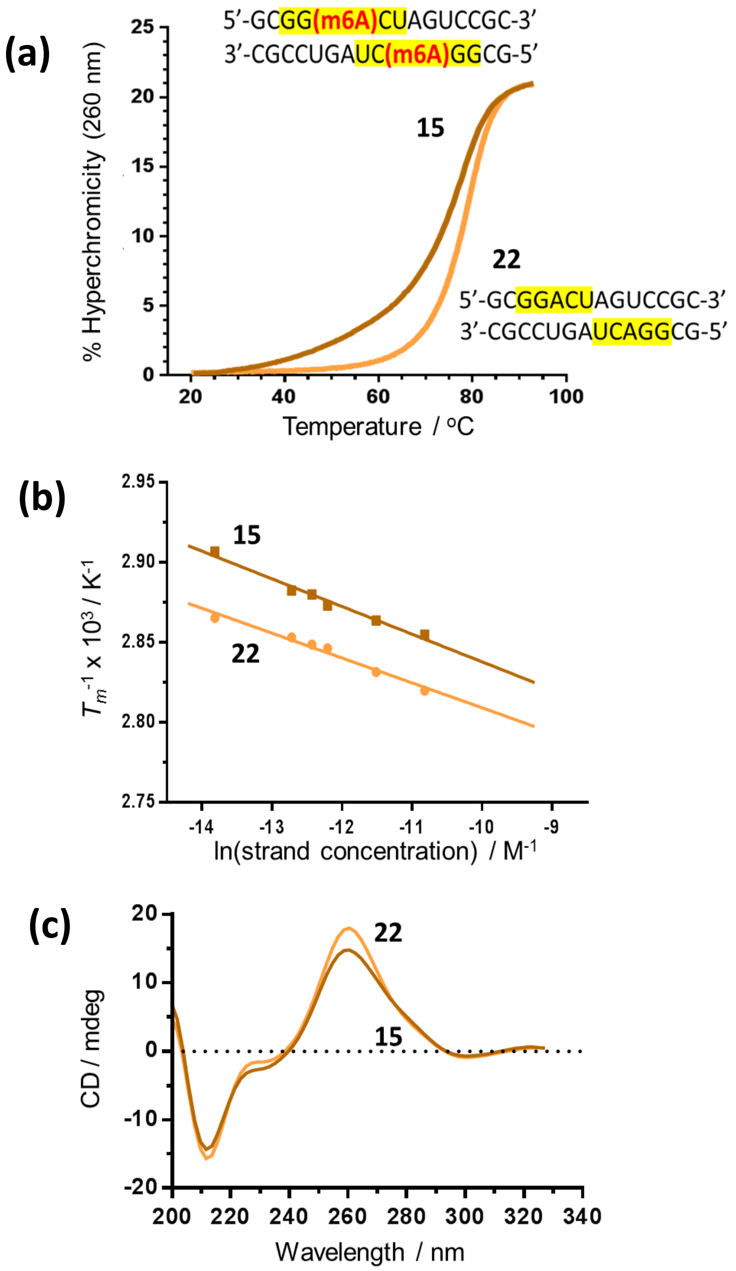

Figure S10. Effects of m6A modification on consensus sequence **15**. (a) UV-melting profiles of **15** and its corresponding unmethylated reference sequence **22** at 5 µM strand concentration in 10 mM sodium phosphate buffer containing 150 mM NaCl, pH 7.4. Both oligos showed monophasic melt profile with strong hyperchromicity, which are indicative of bimolecular duplex structures. (b) Consistent with this result, van't Hoff analyses showed dependence of melting temperatures on strand concentrations for **15** and **22**. (c) Overlay of the CD spectra of **15** and **22** (both at 5 µM strand concentration) indicates no significant overall conformational change in the presence of m6A modification. Both oligos showed characteristic A-form duplex structure.

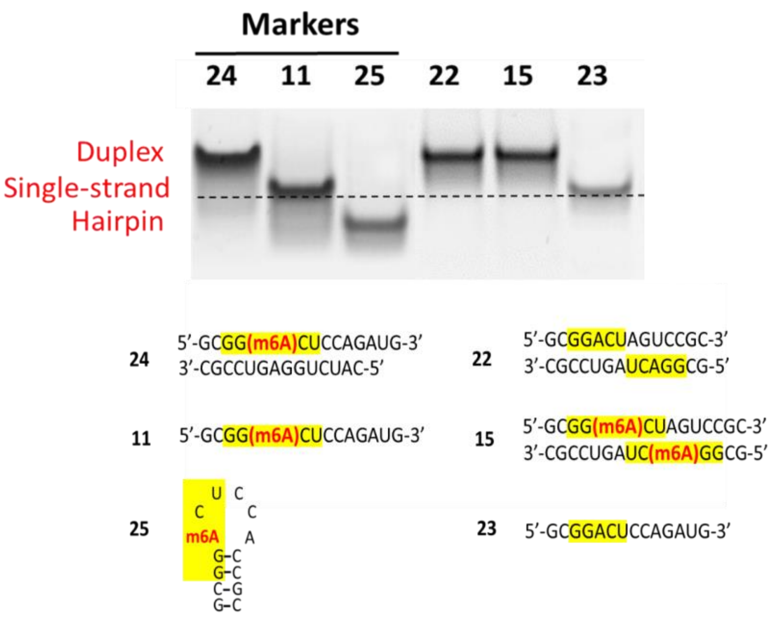

Figure S11. Non-denaturing PAGE analysis of oligos at 5 µM strand concentration showed no evidence of hairpin formation or other major conformational change in **15** on m6A methylation, unlike the context of **19** and **21**. This resulted in little or no demethylation of **15** by FTO and ALKBH5. **11** existed as single strand under the same experimental conditions and is selectively demethylated. The concentration of markers (**24**, **11** and **25)** used was 10 µM.

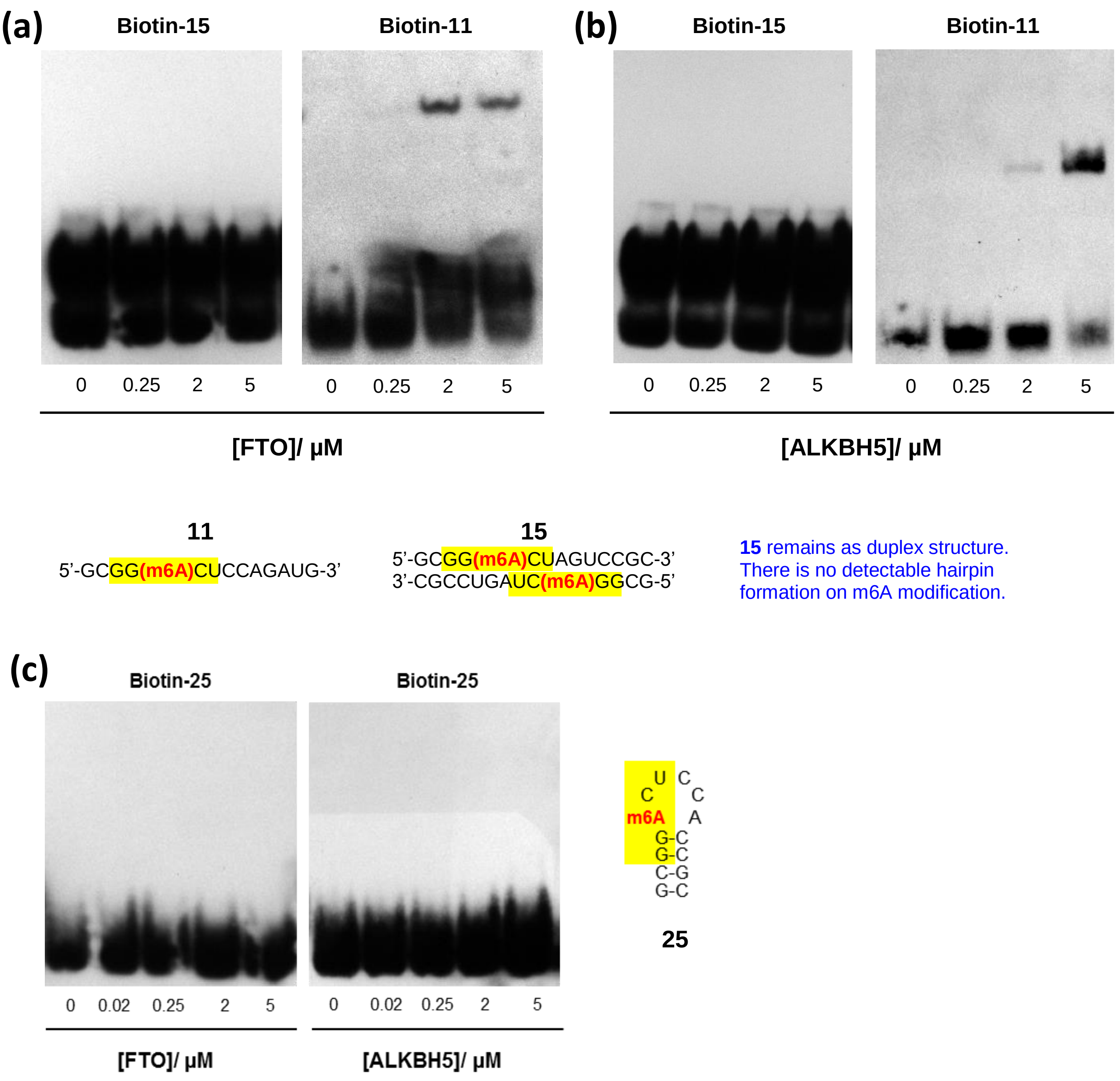

Figure S12. Electrophoretic mobility shift assay (EMSA) of biotin-labelled **11** and **15** (both at 4 nM) with increasing concentrations of m6A-demethylases (a) FTO and (b) ALKBH5, as indicated. Both FTO and ALKBH5 showed significant binding affinity to biotin-**11**, but not to biotin-**15** even though both RNAs contain the same m6A consensus motif. Unlike **19** and **21**, m6A did not induce hairpin formation or other major conformational change in **15**; **11**, on the contrary, exists predominantly as random coil under the same experimental conditions (Supplementary Fig. S10, S11 and S13). Modelling studies indicated that biotin labelling at the 3′-end of **11** and **15** is unlikely to affect their binding affinities substantially. The GG(m6A)CU consensus motifs are highlighted in yellow. (c) Unexpectedly, there was no binding of biotin-**25** to FTO and ALKBH5, despite significant, dose-dependent binding of **25** (unlabelled) to FTO (K_D = 91.3 ± 4.65 µM) and ALKBH5 (K_D = 75.3 ± 2.01 µM), as determined by MST experiments. The biotin label might have prevented the binding of biotin-**25** with both proteins in this sequence context.

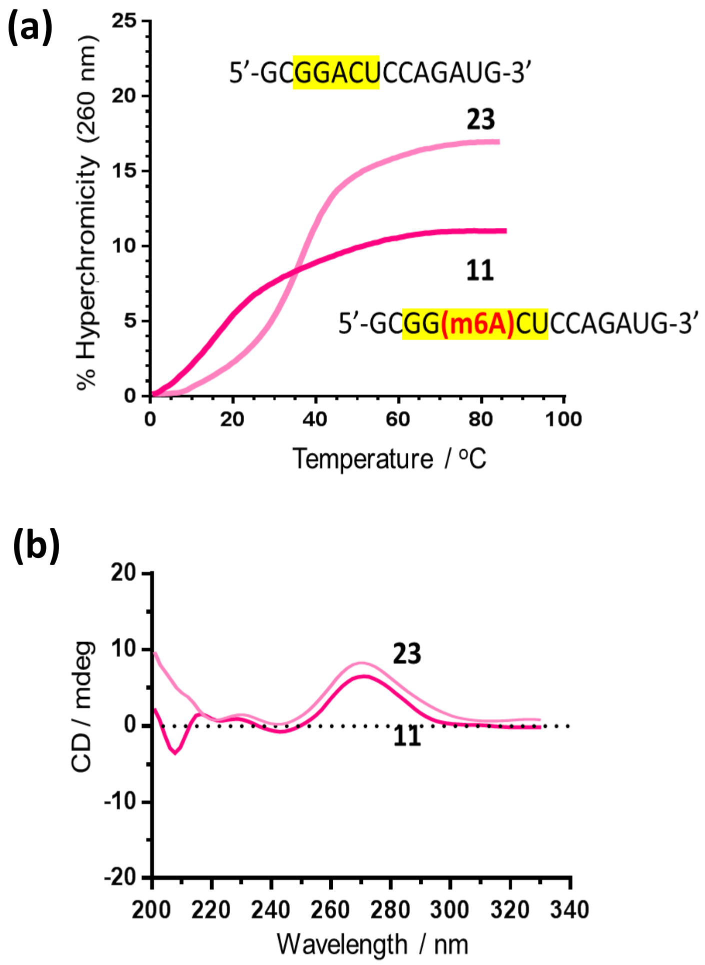

Figure S13. Effects of m6A modification on consensus sequence **11**. (a) UV-melting profiles of **11** and its corresponding unmethylated reference sequence **23** at 5 µM strand concentration in 10 mM sodium phosphate buffer containing 150 mM NaCl, pH 7.4. Both **11** and **23** existed as single-strands. The presence of m6A modification has a destabilising effect on **23** (T_m of **23** reduces from 36.1 °C to 20.3 °C on methylation). (b) Overlay of the CD spectra of **11** and **23** (both at 5 µM strand concentration) indicates no significant overall conformational change in the presence of m6A modification.

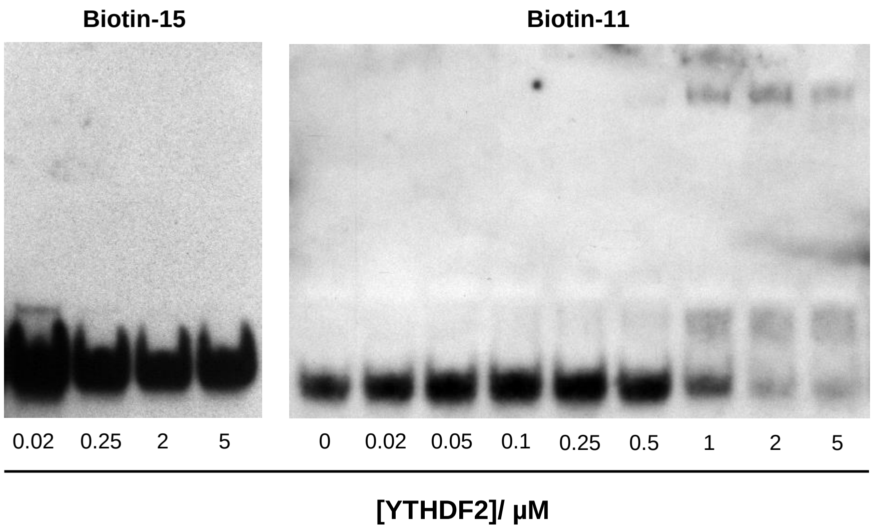

11

5'-GCGG(m6A)CUCCAGAUG-3'

15

5'-GCGG(m6A)CUAGUCCGC-3'
3'-CGCCUGAUC(m6A)GGCG-5'

15 remains as duplex structure. There is no detectable hairpin formation on m6A modification.

Figure S14. EMSA of biotin-labelled **15** and **11** (both at 4 nM) with increasing concentrations of YTHDF2, as indicated. YTHDF2 showed significant binding affinity to biotin-**11**, but not to biotin-**15** even though both RNAs contain the same m6A consensus motif. Modelling studies indicated that biotin labelling at the 3′-end of **11** and **15** is unlikely to affect the binding affinity of YTHDF2 substantially. The GG(m6A)CU consensus motifs are highlighted in yellow.
